# Cortical haemodynamic response measured by functional near infrared spectroscopy during a verbal fluency task in patients with major depression and borderline personality disorder

**DOI:** 10.1016/j.ebiom.2019.11.047

**Published:** 2019-12-24

**Authors:** Syeda F. Husain, Tong-Boon Tang, Rongjun Yu, Wilson W. Tam, Bach Tran, Travis T. Quek, Shi-Hui Hwang, Cheryl W. Chang, Cyrus S. Ho, Roger C. Ho

**Affiliations:** a Institute for Health Innovation and Technology (iHealthtech), National University of Singapore, Singapore; b Department of Psychological Medicine, Yong Loo Lin School of Medicine, National University of Singapore, Singapore; c Centre for Intelligent Signal and Imaging Research (CISIR), University Teknologi PETRONAS, Perak, Malaysia; d Department of Psychology, Faculty of Arts and Social Science, National University of Singapore, Singapore; e Alice Lee Centre for Nursing Studies, Yong Loo Lin School of Medicine, National University of Singapore, Singapore; f Johns Hopkins Bloomberg School of Public Health, Johns Hopkins University, Baltimore, MD, United States; g Institute for Preventive Medicine and Public Health, Hanoi Medical University, Hanoi, Vietnam; h Center of Excellence in Behavioral Medicine, Nguyen Tat Thanh University, Ho Chi Minh City, Vietnam; i Department of Psychological Medicine, National University Health System, Singapore

**Keywords:** Near-infrared spectroscopy, Verbal fluency task, Prefrontal cortex, Haemodynamic response, Borderline personality disorder, Major depressive disorder

## Abstract

**Background:**

Functional near infrared spectroscopy (fNIRS) provides a direct and quantitative assessment of cortical haemodynamic function during a cognitive task. This functional neuroimaging modality may be used to elucidate the pathophysiology of psychiatric disorders, and identify neurophysiological differences between co-occurring psychiatric disorders. However, fNIRS research on borderline personality disorder (BPD) has been limited. Hence, this study aimed to compare cerebral haemodynamic function in healthy controls (HC), patients with major depressive disorder (MDD) and patients with BPD.

**Methods:**

fNIRS signals during a verbal fluency task designed for clinical assessment was recorded for all participants. Demographics, clinical history and symptom severity were also noted.

**Findings:**

Compared to HCs (*n* = 31), both patient groups (MDD, *n* = 31; BPD, *n* = 31) displayed diminished haemodynamic response in the frontal, temporal and parietal cortices. Moreover, haemodynamic response in the right frontal cortex is markedly lower in patients with MDD compared to patients with BPD.

**Interpretation:**

Normal cortical function in patients with BPD is disrupted, but not as extensively as in patients with MDD. These results provide further neurophysiological evidence for the distinction of patients with MDD from patients with BPD.

**Research in Context**

**Evidence before this study**

Borderline Personality Disorder (BPD) affects up to 20% of psychiatric patients, with significant personal and societal costs. Regrettably, the pathophysiology is poorly understood and efforts to discover potential biomarkers is limited. Functional near infrared spectroscopy has been widely applied to other common psychiatric disorders because it is a safe, non-invasive, and economical method of directly assessing haemodynamic response in the cerebral cortex during cognitive tasks. An fNIRS diagnostic paradigm, using a modified verbal fluency task, has been previously designed and validated for clinical settings. It is well established that patients with common psychiatric disorders, such as major depressive disorder (MDD), have lower haemodynamic response in frontal, temporal and parietal cortices, compared to healthy controls (HC). In addition, this diagnostic paradigm differentiates between patient groups, specifically MDD from schizophrenia and bipolar disorder.

**Added value of this study**

Despite numerous reports of the clinical utility of the fNIRS diagnostic paradigm, this protocol has yet to be applied to patients with BPD. Therefore, the objective of this study is to compare the haemodynamic responses of patients with BPD to those of matched HC and patients with MDD. Similar to patients with MDD, those with BPD display marked reduction in haemodynamic response throughout the frontal, temporal and parietal cortices, albeit to a lesser degree. Moreover, patients with MDD had significantly lower haemodynamic response in the right frontal gyrus that patients with BPD.

**Implications of all the available data**

Results from this study are in line with previous reports of abnormalities in patients with BPD observed with other common neuroimaging modalities and molecular methods. fNIRS in particular, can detect neurophysiological disruptions in patients with BPD, which is unique to both healthy and patient controls. Still, large scale studies are needed to establish the utility of fNIRS for the differential diagnosis of BPD.

Alt-text: Unlabelled box

## 1 Introduction

Borderline personality disorder (BPD) is a serious psychiatric disorder characterised by a pervasive pattern of unstable emotional regulation, interpersonal relationships, self-image and impulse control [1]. In community samples, BPD prevalence is approximately 1%, while estimates for psychiatric samples range from 10% to 20% [2]. Suicide attempts and self-harming are concerning and common behaviours for this patient group. Thus, patients with BPD are high utilisers of emergency psychiatric services. Other typically associated behaviours include reckless driving, domestic violence, imprisonment and pathological gambling [3]. Therefore, timely diagnosis and appropriate treatment of BPD is essential, to mitigate personal losses and societal burden [4].

A diagnosis of BPD is established when 5 out of 9 criteria defined in the fifth edition of the Diagnostic and Statistical Manual of Mental Disorders (DSM-5) are present [1]. Despite these guidelines, this metal disorder remains under-recognised [4] because of symptom heterogeneity within this patient group [5] and overlapping symptoms with mood disorders [6]. Up to 85% of patients with BPD meet the criteria for major depressive disorder (MDD) [7], and fewer patients with BPD experience remission compared to those with MDD alone [8]. This may be because patients with BPD tend to overreport their depressive symptoms [9]. Hence, their depressive symptoms usually do not improve without first addressing the underlying personality disorder [10]. These diagnostic challenges are further compounded by the misrepresentation of BPD as being difficult to manage [11]. Hence, psychiatrists may prefer to focus on co-occurring MDD, which they may believe to be more a more manageable disorder [4]. However, treatment strategies for BPD and MDD differ. In addition to antidepressants, patients with BPD benefit from psychotherapy, second-generation antipsychotics and mood stabilisers [12,13].

Given the limitations of psychiatric nosology, laboratory or imaging tests to aid in the differential diagnosis of BPD from mood disorders are needed [5]. Although such tests are currently not available, findings from neuroimaging research are encouraging [14]. For example, a recent voxel-based meta-analysis showed that BPD and bipolar disorders differs in grey matter volume pattern and grey matter density alteration, suggesting that these disorders are not on the same affective spectrum [15]. Similarly, neuroanatomical and neurophysiological differences between BPD and MDD have been detected using magnetic resonance imaging (MRI) [16,17], positron emission tomography (PET) [18], electroencephalography (EEG) [19] and Functional near infrared spectroscopy (fNIRS) [20,21] techniques. Amongst these techniques, fNIRS is an emerging functional neuroimaging modality which may be particularly suited as a diagnostic tool for psychiatric disorders. fNIRS is used to study neurophysiology as this technology can continually monitor haemodynamic changes in the cerebral cortex using near-infrared light [22]. Wavelengths of near-infrared light have the unique property of passing through tissues until it reaches the cortex, where it is preferentially absorbed by oxy-haemoglobin and deoxy-haemoglobin [23]. fNIRS signals are believed to reflect the underlying neuronal activity, described in a phenomenon known as neurovascular coupling [24]. Upon regional neuronal activity, the increase in blood flow and volume is several folds higher than the metabolic demands, resulting in a nett increase in oxy-haemoglobin and a simultaneous slight decrease in deoxy-haemoglobin [25]. Although fNIRS can only measure cortical regions, it is safe, non-invasive, non-restrictive, quiet and tolerant to motion. Therefore, it is often used for the direct observation of haemodynamic changes in psychiatric patients during cognitive tasks [26].

The verbal fluency task (VFT) has been adopted for fNIRS research, as the conventional VFT is frequently used by clinicians to evaluate frontal lobe function in neuropsychiatric patients [27]. While fNIRS publications vary in their VFT design and fNIRS signal processing, the protocol proposed by Takizawa et al. [28,29] was developed specifically for clinical settings. It has been extensively validated on common psychiatric disorders, including MDD [30], [31], [32]. However, fNIRS studies on BPD are numbered, comparing patients with BPD only to HC during emotional tasks [20,21]. Hence, the aim of this study was to compare fNIRS signals during the VFT between HC, patients with MDD and patients with BPD. We hypothesise that the mean oxy-haemoglobin changes in the frontal, temporal and parietal cortices is the highest in HC, followed by patients with BPD and is the lowest in patients with MDD.

## 2 Methods

### 2.1 Participants

Thirty-one patients with BPD, 31 patients MDD and 31 HC who were between 21–65 years old were included in this study (Age in years: BPD, 31.8 ± 10.2; MDD, 31.8 ± 10.1; HC, 31.7 ± 10.5). All participants were female because these disorders are female predominant [1] and study participants were homogeneous by gender. Across the 3 groups, subjects were matched for age, ethnicity and years of education. Patients were recruited from the outpatient psychiatric clinic at the National University Hospital, Singapore, while HC were recruited from the community. Each patient had been diagnosed by a psychiatrist, according to the DSM-5 [1] for MDD or BPD, using the Structured Clinical Interview for the DMS-5 [33]. Individuals were excluded from the study if they had conditions that could affect the central nervous system, including cerebrovascular diseases, respiratory diseases, hepatic diseases, kidney diseases, cancer, epilepsy or intellectual disability. HC who reported past psychiatric history, HC and patients who received psychotherapy and participants who reported drowsiness on the day of participation were excluded. Psychosocial functioning and depressive symptoms for each participant were evaluated using the global assessment of functioning (GAF) [34] and 17-item Hamilton rating scale for depression (HAM-D) [35], respectively. HC with a HAM-D score of 8 or higher were also excluded [36]. In addition, borderline personality traits amongst patients was assessed using the borderline personality questionnaire (BPQ) [37].

Study details were fully explained to participants, and their written informed consent was obtained. The authors assert that all procedures contributing to this work comply with the ethical standards of the Declaration of Helsinki, and the ethical principles in the Belmont Report. It was approved by the Domain Specific Review Board of the National Healthcare Group, Singapore (protocol number 2017/00509).

### 2.2 Verbal fluency task

Prior to the fNIRS measurement recording, participants watched a demonstration video, in which they were asked to remain seated, avoid excessive body or head movements, and focus on a cross displayed during the VFT. The paradigm used in previous studies [29] was modified for the English language (Supplementary Fig. 1). It consisted of a 30 s pre-task period, 60 s task period, and a 70 s post-task period. During the pre and post-task periods, participants were asked to say “A, B, C, D, E” aloud and repeatedly. During the task period, they were instructed to generate as many words as possible, beginning with A, F and S for 20 s per letter. The total number of unique words, enunciated within the task period, was recorded as the task performance. Before the actual trial, participants were asked to practice the VFT for a shorter duration, and with the letters H, B and P. This ensured all participants understood the task and responded to the cues correctly during the actual trial.

### 2.3 fNIRS measurement

A 52-channel fNIRS system (ETG-4000. Hitachi Medical Co., Tokyo, Japan) measured relative oxy-haemoglobin and deoxy-haemoglobin changes using 2 NIR light wavelengths (695 and 830 nm) [38]. Emitter and detector optodes were arranged 3 cm apart. The area between each emitter and detector pair is called a channel. Anatomically, channels correspond to cortical regions 2–3 cm beneath the skin and scalp surface [39]. Optodes were placed on the forehead and scalp, with the lowest optodes placed along the T4-Fpz-T3 line, defined by the 10/20 system. This arrangement allowed for haemoglobin changes in the bilateral prefrontal cortex, frontopolar cortex, and the anterior regions of the superior and middle temporal cortices to be measured. These approximate channel locations are based on the anatomical craniocerebral correction of the international 10/20 system.

### 2.4 fNIRS signal analysis

fNIRS signals were processed according to the method described by Takizawa et al. [29]. Oxy-haemoglobin, deoxy-haemoglobin and total haemoglobin were derived from optical densities using the modified Beer-Lambert law. Haemoglobin changes during the task period were normalised by linear fitting between a 10 s baseline at the end of the pre-task period, and a 5 s post-task baseline period that is 50 s into the post-task period (Supplementary Fig. 1). A moving average factor of 5 was applied to remove short term motion artefacts. An algorithm identifying channels with body movement artefacts, or high and low frequency noise was applied. Artefact channels were removed from further analysis, while the mean oxy-haemoglobin and deoxy-haemoglobin changes during the pre-task and task periods at each remaining channel was determined for each subject. Since changes in oxy-haemoglobin are larger than deoxy-haemoglobin [40], results for the latter are reported in supplementary materials.

**Fig. 1:**
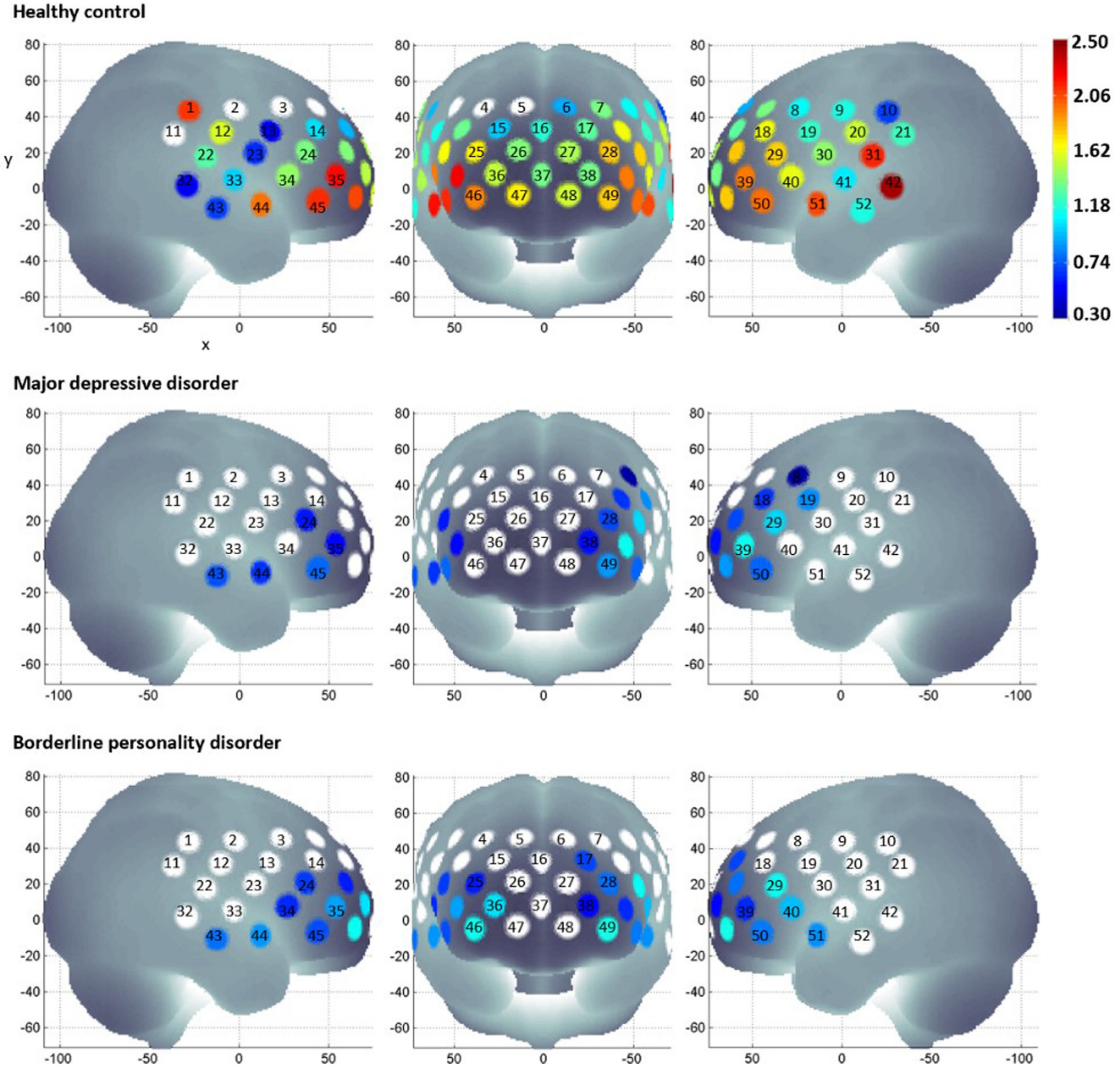
Activation at each channel was determined by paired sample *t*-test comparing the mean oxy-haemoglobin during the pre-task baseline period and task period. The effect size of activation during the VFT is indicated by the colour gradient. Channels that did not show statistically significant differences in oxy-haemoglobin between the pre-task baseline and task periods are in white.

### 2.5 Statistical analysis

To determine if activation during the VFT occurs at each channel for each diagnostic group, Student's paired *t*-test was used to compare mean oxy-haemoglobin during the pre-task baseline period and task period. To correct for multiple comparisons, a maximum false discovery rate of 0.05 (two-tailed) was applied such that false positive results were limited to 5% [41].

The effect of diagnostic group on categorical variables were determined using chi-square test. Student's *t*-test or one-way analysis of variance (ANOVA) with post-hoc Fisher's least significant difference test was used to determine the effect of diagnostic group on continuous variables. Categorical variables are gender, ethnicity, handedness, family psychiatric history, past admission to psychiatric ward and treatment with psychotropic drugs. Psychotropic drugs were further classified into antidepressants, anxiolytics and sedatives, antipsychotics and mood stabilisers (Supplementary Table 1). Continuous variables are age, years of education, GAF score, HAM-D score, BPQ score, number of words generated, number of available channels, mean oxy-haemoglobin at each channel, age at psychiatric illness onset, duration of psychiatric illness and equivalent doses of antidepressants, anxiolytics and sedatives, as well as antipsychotics. Equivalent doses were calculated based on published mean dose ratios. Reference drugs for each class are fluoxetine, diazepam and chlorpromazine, respectively [42,43]. For patients receiving more than one drug in each class, the combined equivalent dose was calculated. When mean oxy-haemoglobin at any channel differed between patient groups, subsequent regression analysis was carried out, with mean oxy-haemoglobin as the dependant variable. Independent variables included in the model were diagnosis and any other demographic, clinical or behavioural variables that differed between patient groups.

All tests were two-tailed, with a significance level of *p* < 0.05. Data are expressed as mean and standard deviation. Wherever a difference in mean oxy-haemoglobin was observed between groups, the effect size (Hedge's *g*) was reported or used in figures. Statistical analysis was done on SPSS Statistic 21.0 (IBM). Channel positions were plotted using NFRI functions toolbox [44,45].

## 3 Results

### 3.1 Demographic and clinical data

HC, patients with MDD and patients with BPD did not differ in age (HC, 31.7 ± 10.5 years; MDD, 31.8 ± 10.1 years; BPD, 31.8 ± 10.2 years), ethnicity, handedness, years of education, number of words generated and family psychiatric history (*p* > 0.05; Table 1). Unsurprisingly, HC had higher GAF scores [*F* = 73, *p* ≤ 0.001] and lower HAM-D scores [*F* = 45.4, *p* ≤ 0.001] than patients with MDD [GAF: *g* = 2.66, *p* ≤ 0.001, 95% CI, (21.6 to 32.6); HAM-D: *g* = 2.53*,p* ≤ 0.001, 95% CI, (10.1 to 16.7)] and patients with BPD [GAF: *g* = 3.08, *p* ≤ 0.001, 95% CI, (25.1 to 36.2); HAM-D: *g* = 2.23*,p* ≤ 0.001, 95% CI, (10.7 to 17.3)]. Patient groups did not differ in their GAF scores, HAM-D scores, age at onset, duration of illness, and number of patients on pharmacotherapy. Specifically, patient groups did not differ in the number of patients on antidepressants, anxiolytics and sedatives and antipsychotics, as well as equivalent doses for these drug classes (*p* > 0.05). However, compared to patients with MDD, patients with BPD had higher BPQ scores [*t* = 4.5, *df* = 52.5, *g* = 1.1, *p* ≤ 0.001, 95% CI, (6.6. to 17.5)], higher past admissions to psychiatric ward [*X*^2^(2, *n* = 62) = 9.3, *p* = 0.005] and a larger number of patients on mood stabilisers [*X*^2^(2, *n* = 20) = 4.3, *p* ≤ 0.001].

**Table 1:** Demographic and clinical characteristics.

	HC (*n* = 31)	MDD (*n* = 31)	BPD (*n* = 31)	*p*-value
Age (years)	31.7 ± 10.5	31.8 ± 10.1	31.8 ± 10.2	0.142
Ethnicity				0.993
Chinese	24 (77.4%)	25 (80.6%)	23 (74.2%)	
Malay	4 (12.9%)	3 (9.68%)	4 (12.9%)	
Indian	2 (6.45%)	2 (6.45%)	2 (6.45%)	
Others	1 (3.23%)	1 (3.23%)	2 (6.45%)	
Handedness^a^				0.779
Right	27 (93.1%)	19 (90.5%)	19 (90.5%)	
Left	1 (3.4%)	2 (9.5%)	1 (4.8%)	
Ambidextrous	1 (3.4%)	0	1 (4.8%)	
Education (years)	15.9 ± 2.1	15.1 ± 2.3	14.8 ± 2.3	0.142
Number of words generated	19 ± 6	17.2 ± 6.2	15.7 ± 5.3	0.091
Number of available channels	37.1 ± 10.2	40.5 ± 7.5	37.3 ± 9.4	0.278
Family psychiatric history^a^	3 (11.5%)	7 (24.2%)	8 (30.8%)	0.237
GAF score	94.2 ± 7.7	67.1 ± 12.2	62.6 ± 12.3	**≤0.001**^b^
HAM-D score	2.4 ± 2.2	15.7 ± 7.1	16.4 ± 8.6	**≤0.001**^b^
BPQ score	–	39.7 ± 12.5	51.7 ± 8.4	**≤0.001**
Age at onset (years)^a^	–	27.2 ± 9.6	23.9 ± 8.9	0.178
Duration of illness (years)^a^	–	4.6 ± 5.3	7.5 ± 6.6	0.068
Past admission to psychiatric ward	–	9 (29%)	21 (67.7%)	**0.005**
Pharmacotherapy	–	22 (71%)	26 (83.9%)	0.211
Antidepressants	–	21 (67.7%)	24 (77.4%)	0.570
Anxiolytics & sedatives	–	5 (16.1%)	5 (16.1%)	1
Antipsychotics	–	2 (6.5%)	8 (25.8%)	0.081
Mood stabilisers	–	1 (3.2%)	17 (54.8%)	**≤0.001**
Fluoxetine eq. dose (mg/day)	–	27.1 ± 14.9	34.2 ± 17.3	0.146
Diazepam eq. dose (mg/day)	–	5.5 ± 4.4	9.9 ± 7.2	0.277
Chlorpromazine eq. dose (mg/day)	–	175.8 ± 34.3	192 ± 128.2	0.869

Mean ± SD are shown and *p*-values <0.05 are in bold.

a Complete demographic and clinical data were not obtained for all subjects (Known handedness in healthy controls, *n* = 29; in major depressive disorder, *n* = 21; in borderline personality disorder, *n* = 21. Known family history of psychiatric illness in healthy controls, *n* = 26; in major depressive disorder, *n* = 29; in borderline personality disorder, *n* = 26. Known age at onset and duration of illness in major depressive disorder, *n* = 31; in borderline personality disorder, *n* = 30).

b Post-hoc test showed statistically significant differences in GAF and HAM-D scores between healthy controls and patients with major depressive disorder (*p* ≤ 0.001) and between healthy controls and patients with borderline personality disorder (*p* ≤ 0.001), but not between patient groups (GAF, *p* = 0.200; HAM-D, *p* = 0.699).

### 3.2 Haemodynamic response during the VFT

There were no differences in the number of available channels between the 3 diagnostic groups (*p* > 0.05, Table 1). Oxy-haemoglobin increase during the task period, relative to the pre-task baseline period, was observed in 48 channels for HC (*p*-values from ≤0.001 to 0.038; Fig. 1). In other words, activation amongst HCs occurs in much of the frontal, temporal and parietal cortices. Moreover, there is a simultaneous, albeit smaller, decrease in deoxy-haemoglobin in several of these channels for HCs (Supplementary Fig. 2). In contrast, oxy-haemoglobin increase from pre-task baseline occurs in 14 channels for patients with MDD (*p*-values from ≤0.001 to 0.035) and 18 channels for patients with BPD (*p*-values from ≤0.001 to 0.044; Fig. 1). Activation in patient groups is limited to the right and left middle frontal, right and left inferior fontal, right superior temporal, right middle temporal and right inferior temporal gyri. In addition, the left superior temporal gyrus was activated in patients with BPD. Even so, oxy-haemoglobin increase from baseline in these regions are smaller for patient groups compared to HC. Furthermore, decrease in deoxy-haemoglobin occurs in a handful of channels in the middle and inferior frontal gyrus for patients with MDD, and in the superior, middle and inferior formal gyrus for patients with BPD (Supplementary Fig. 2).

**Fig. 2:**
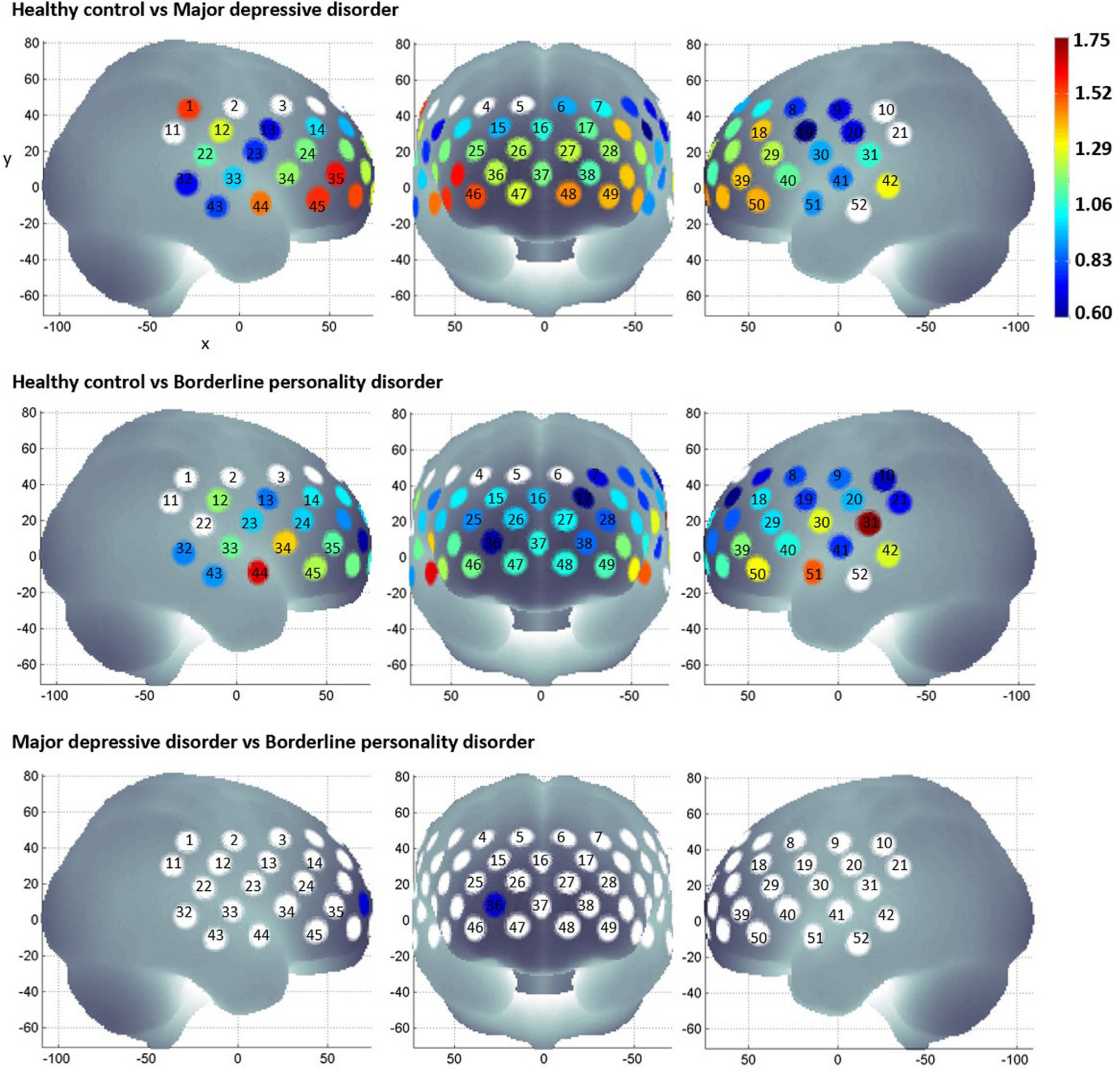
Group differences in mean oxy-haemoglobin during the VFT was determined by one-way ANOVA with post-hoc Fisher's least significant difference test. The effect size of group differences are indicated by the colour gradient. Channels that did not show statistically significant differences between the 3 groups are white.

Compared to HC, patients with MDD and BPD have lower mean oxy-haemoglobin during the task period in 44 channels (*p*-values from ≤0.001 to 0.049) and 43 channels (*p*-values from ≤0.001 to 0.037), respectively (Fig. 2). For both patient groups, these channels are located at the right and left precentral, right and left postcentral, right and left superior frontal, right and left middle frontal, right and left inferior frontal, right and left superior temporal as well as the right and left middle temporal gyri. In addition, reduced mean oxy-haemoglobin during the task period compared to HC is observed in the right supramarginal gyrus of patients with MDD, while in patients with BPD, it is observed in the left supramarginal gyrus.

**Fig. 3:**
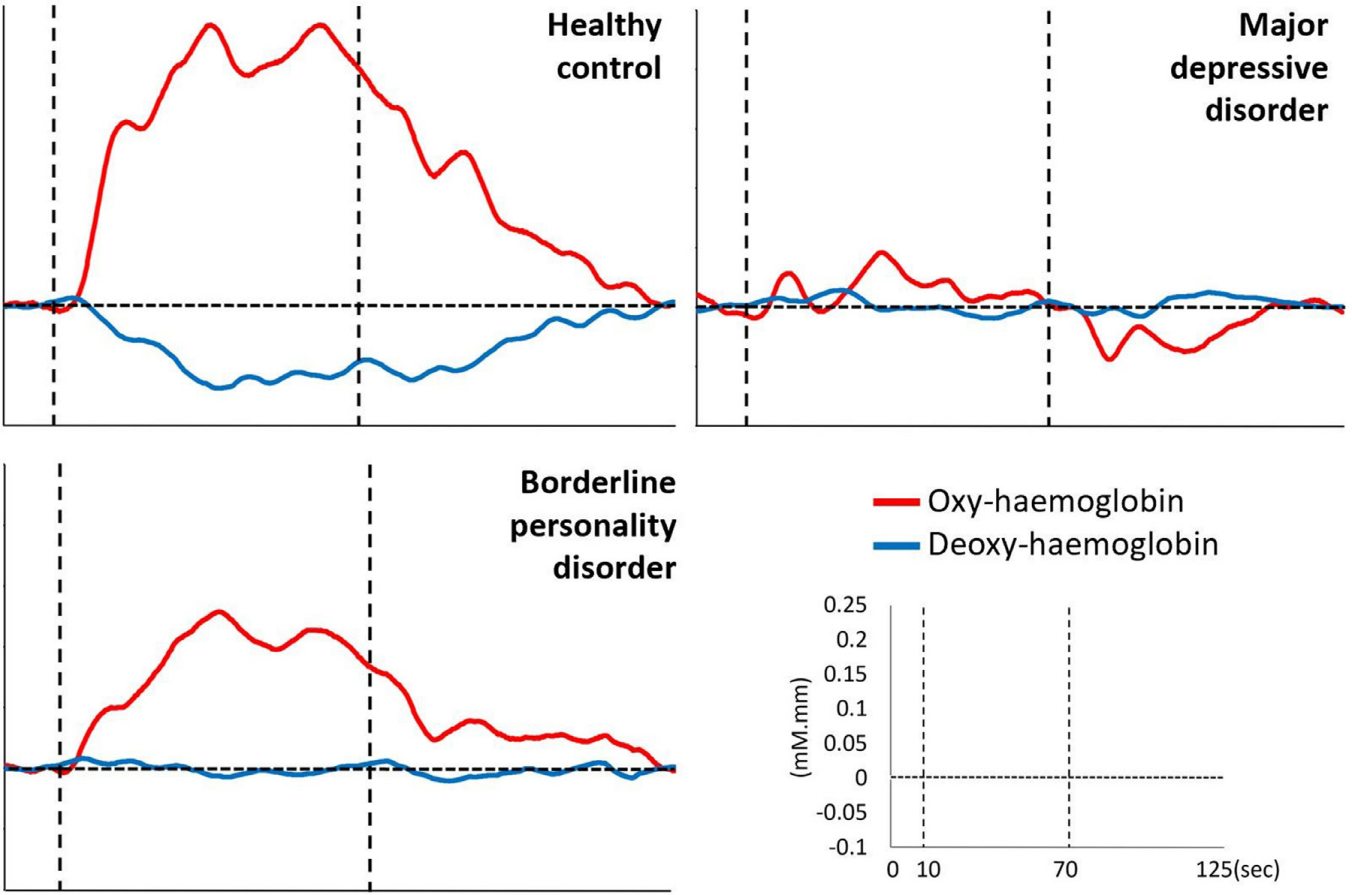
Average oxy-haemoglobin waveforms at channel 36.

When mean oxy-haemoglobin during the task period are compared between patient groups only, patients with MDD have lower haemodynamic response at channel 36 of the right middle frontal gyri compared to patients with BPD (*p* = 0.027; Fig. 3). Subsequent linear regression (adjusted *R*^2^ = 0.083) showed that diagnosis is associated with mean oxy-haemoglobin (β = 0.092, S.E. = 0.033, *p* = 0.007), but not past admission to psychiatric ward (β = (−0.029), S.E. = 0.029, *p* = 0.323) or BPQ score (β = (−0.001), S.E. = 0.001, *p* = 0.539). Although exploring the relationship between mean oxy-haemoglobin at channel 36 and clinical or behavioural variables within diagnostic groups was not an aim of this study, Pearson's correlation tests suggest these variables are not associated with haemodynamic response (Supplementary Table 2).

## 4 Discussion

The present fNIRS study suggests that haemodynamic dysfunction during the VFT occurs in the frontal, temporal and parietal cortices of patients with BPD. While diminished activation compared to HCs has been reported for common psychiatric disorders by several authors [30], [31], [32], this is the first time an fNIRS protocol, designed for clinical settings [29], has been applied to patients with BPD. Furthermore, cortical haemodynamic dysfunction in the right middle frontal gyrus is less severe in patients with BPD than patients with MDD, despite similar GAF and HAM-D scores in both patient groups, and higher past admission rates and BPQ scores in patients with BPD. The difference in fNIRS signals between BPD and MDD is further supported by differences in cortical regions detected by MRI [16,17], EEG [19] and PET [18] techniques. This intermediate cortical activation may reflect the time course of BPD, namely persistent functional impairment despite remission, but lower rates of relapse compared to MDD alone [8,46].

The pathophysiology of BPD is believed to be multifaceted, involving psychosocial, genetic and neurobiological factors [47]. A growing number of reports suggest abnormalities in the endocrinology, neurochemistry, neuroanatomy and neurophysiology of patients with BPD. Endocrinological alterations include hypothalamic pituitary adrenal axis dysfunction [48], [49], [50], [51], reduction in peripheral oxytocin levels [52,53] and elevated peripheral testosterone levels [54]. Like other common psychiatric disorders, cerebral monoamine dysregulation is also apparent in BPD. Specifically, serotonergic and dopaminergic abnormalities have been identified using PET [55,56] and molecular genetics [57]. Structural neuroimaging techniques have shown reductions in the hippocampus, amygdala [58] and cortical regions of the frontal [59], parietal [60] and temporal lobes [61]. At the same time, functional neuroimaging approaches such as functional magnetic resonance imaging (fMRI) reveal a hyperactive amygdala following negative external stimuli [62]. Likewise, PET studies have associated several BPD traits with altered glucose metabolism in the amygdala [63] and frontal lobe [64]. Furthermore, altered frontal EEG signals are associated with childhood trauma, dissociative symptoms [65] and impaired emotional processing in patients with BPD [66]. Taken together, concurring evidence derived from various biological techniques, including fNIRS, support a biological model of BPD [67].

Biological technologies that can probe the neurophysiological alterations in BPD, including fNIRS, have improved out understanding of its aetiology and validates the diagnostic criteria of BPD [5,68]. Yet, these technologies have not been introduced in clinical practice [5]. Instead, the available diagnostic tools are structured or semi-structured interviews, and neuropsychiatric questionnaires [5], but these instruments have their limitations as well. During clinical interviews, BPD patients may report feelings of emptiness, which is typically not present in patients with MDD. Yet, the experience is difficult to describe and lacks specificity for the diagnosis of BPD [69]. BPD patients often experience more frequent depressive episodes than patients with MDD alone [70], but self-rated depressive scores amongst BPD patients with and without depression are largely indistinguishable from patients with MDD [71]. Similarly, neuropsychiatric tests assessing memory, attention, executive and visuospatial functions do not detect differences between BPD and MDD [72]. Moreover, these instruments are time consuming and often require a specialist to administer. Consequently, they are not routinely used in clinical settings either [5]. Therefore, further research and development of technologies that may improve clinical practice, such as fNIRS, are necessary [22].

This study is limited by a small sample size. Hence, patients with BPD were not subtyped into those with and without current MDD. Secondly, BPD onset usually occurs in adolescence, but only adults were recruited for this study. Research on the presentation, course and treatment of BPD in adolescents may lead to earlier diagnosis, timely intervention and improved outcomes [5]. Hence, fNIRS studies on adolescent population may enhance our knowledge of BPD pathophysiology. Moreover, a prospective study on adolescents may establish a causal relationship between BPD onset and haemodynamic dysfunction, which could not be established in this cross-sectional study. Thirdly, compared to patients with MDD, a significant proportion of patients with BPD were on mood stabilisers. However, we could not study the relationship between cortical activity and different mood stabilisers or dosages. Future fNIRS studies comparing subgroups of BPD patients on different mood stabilisers may contribute to our understanding of drug mechanisms. Though beyond the scope of this study, future fNIRS studies on male patients with BPD may be of interest [73]. Similarly, fNIRS may identify differences between BPD and other psychiatric disorders with overlapping features, namely anxiety [74], bipolar disorder [75] and psychotic [76] disorders.

In conclusion, findings from this study provide preliminary evidence for future research on functional neuroimaging biomarkers for BPD. fNIRS signals amongst patients with BPD deviate from both healthy individuals and patients with major depression alone. Since fNIRS signals are a direct and objective measure of cortical physiology, these observations lend further support for a neurobiological basis of BPD.

## Funding

No external funding was received for this work. Funders did not have any role in the study design, data collection, data analysis, interpretation, or writing of the report.

## Data availability

The data that support the findings of this study are available on request from the corresponding author. The data are not publicly available due to privacy or ethical restrictions.

## Declaration of competing interest

None.

## Appendix Supplementary materials

Image, application 1
