# Supplementary material for: Cortical haemodynamic response measured by functional near infrared spectroscopy during a verbal fluency task in patients with major depression and borderline personality disorder
Source: eBioMedicine. 2019 Dec 24;51:102586. doi: 10.1016/j.ebiom.2019.11.047 (PMC6938854; doi:10.1016/j.ebiom.2019.11.047)
Supplement: Supplementary file 1 [file mmc1.docx]

**Supplementary materials**


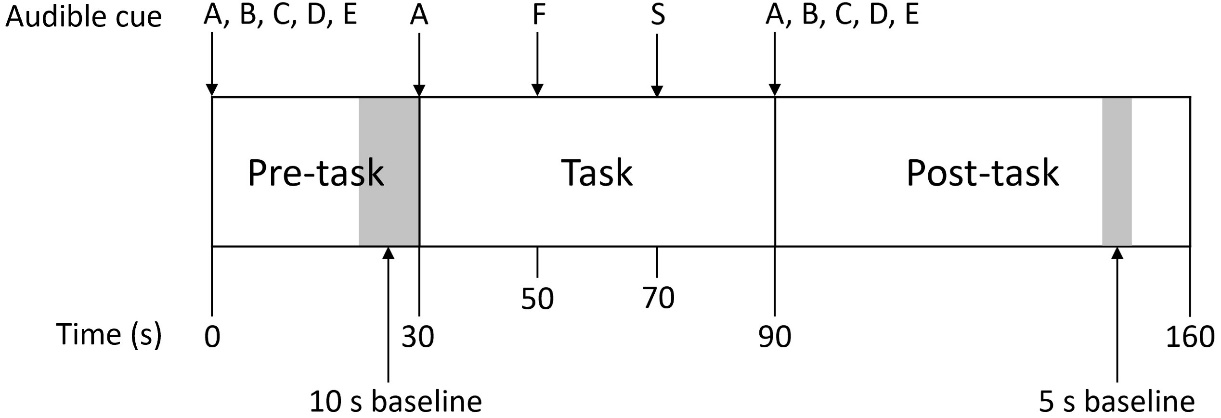


**Supplementary Fig 1.** The verbal fluency task protocol.

**Supplementary table 1.** Psychotropic medication details.

|  | MDD | | BPD | |
| --- | --- | --- | --- | --- |
|  | n | Dose (mg/day) | n | Dose (mg/day) |
| Antidepressants |  |  |  |  |
| *Selective serotonin reuptake inhibitors* |  |  |  |  |
| Escitalopram | 0 | - | 1 | 20 |
| Fluoxetine | 4 | 20 ± 0 | 7 | 28.6 ± 10.7 |
| Fluvoxamine | 7 | 64.3 ± 24.4 | 9 | 111.1 ± 54.7 |
| Paroxetine | 2 | 18.8 ± 8.8 | 1 | 12.5 |
| Sertraline | 4 | 87.5 ± 47.9 | 1 | 100 |
| *Serotonin and norepinephrine reuptake inhibitors* |  |  |  |  |
| Duloxetine | 0 | - | 1 | 6 |
| Venlafaxine | 0 | - | 2 | 112.5 ± 53 |
| *Noradrenergic and specific serotonergic antidepressant* |  |  |  |  |
| Mirtazapine | 3 | 20 ± 8.7 | 3 | 15 ± 0 |
| *Dopamine Norepinephrine Reuptake Inhibitor* |  |  |  |  |
| Bupropion | 1 | 150 | 2 | 150 ± 0 |
| *Tricyclic Antidepressants* |  |  |  |  |
| Amitriptyline | 1 | 25 | 0 | - |
| *Other antidepressants* |  |  |  |  |
| Agomelatine | 2 | 50 ± 0 | 4 | 25 ± 0 |
| Vortioxetine | 1 | 10 | 1 | 10 |
| *Combination antidepressants* |  |  |  |  |
| Agomelatine & bupropion | 1 |  | 1 |  |
| Agomelatine & duloxetine | 0 |  | 1 |  |
| Agomelatine & sertraline | 1 |  | 0 |  |
| Agomelatine, fluoxetine & vortioxetine | 0 |  | 1 |  |
| Amitriptyline & fluvoxamine | 1 |  | 0 |  |
| Bupropion, fluvoxamine & venlafaxine | 0 |  | 1 |  |
| Escitalopram & mirtazapine | 0 |  | 1 |  |
| Fluvoxamine & mirtazapine | 0 |  | 1 |  |
| Mirtazapine & paroxetine | 1 |  | 0 |  |
| Anxiolytics and sedatives |  |  |  |  |
| Alprazolam | 2 | 0.2 ± 0.1 | 1 | 0.5 |
| Clonazepam | 2 | 0.5 ± 0 | 1 | 1 |
| Lorazepam | 0 | - | 2 | 0.8 ± 0.4 |
| Zopiclone | 1 | 7.5 | 2 | 15 ± 0 |
| *Combination anxiolytics and sedatives* |  |  |  |  |
| Alprazolam & zopiclone | 0 |  | 1 |  |
| Antipsychotics |  |  |  |  |
| Olanzapine | 1 | 5 | 1 | 2.5 |
| Paliperidone | 0 | - | 1 | 3 |
| Quetiapine | 1 | 100 | 5 | 150 ± 100 |
| Risperidone | 0 |  | 1 | 1 |
| Mood stabilisers |  |  |  |  |
| Lamotrigine | 0 | - | 3 | 100 ± 50 |
| Sodium valproate | 1 | 300 | 10 | 440 ± 298.9 |
| Topiramate | 0 | - | 4 | 75 ± 50 |


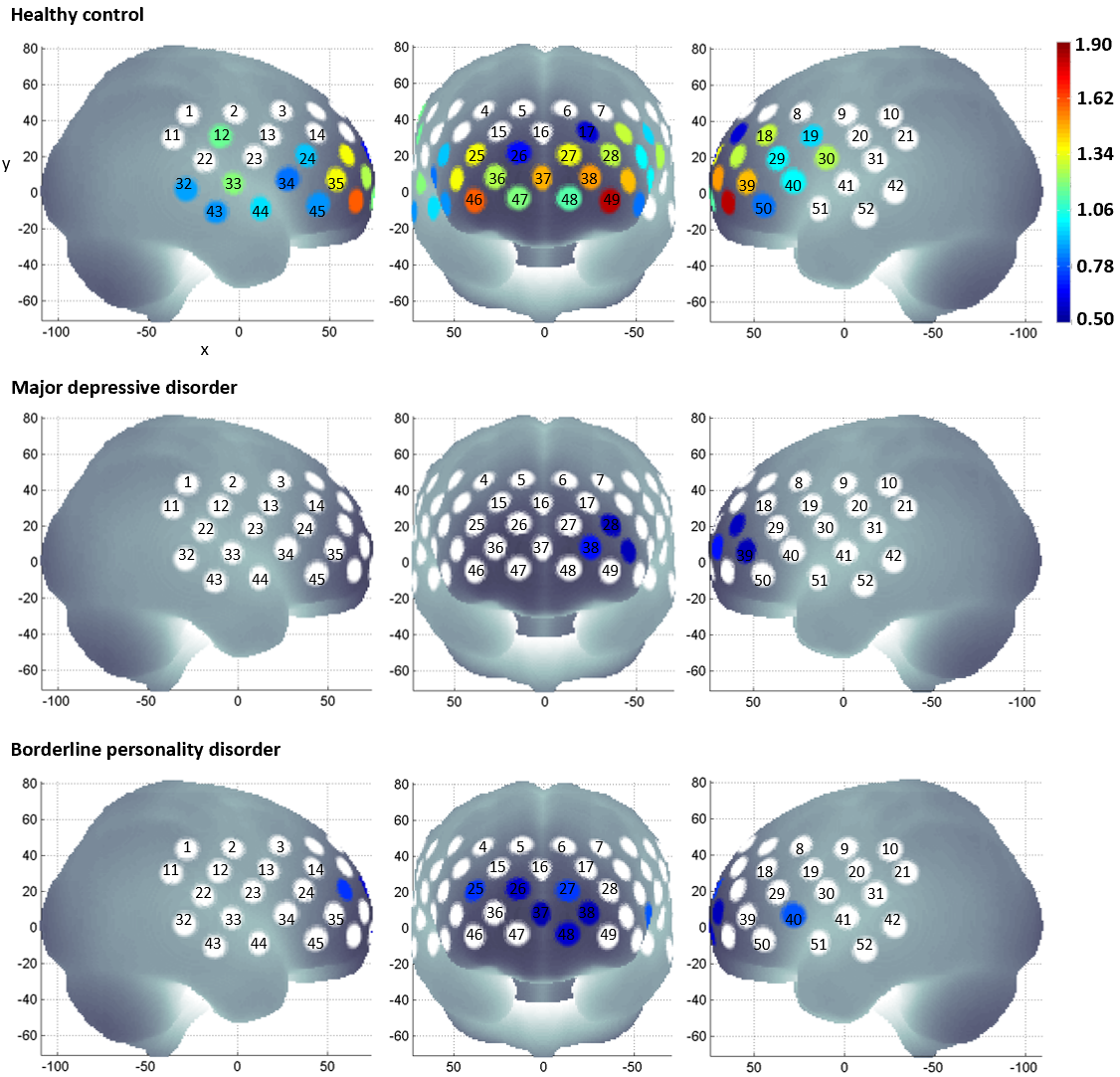


**Supplementary Fig 2.** Comparison of mean deoxy-haemoglobin during the pre-task baseline period and task period using paired t-test. The effect size of activation during the VFT is indicated by the colour gradient. Statistically significant decrease in deoxy-haemoglobin was observed in 29 channels for HC (*p*-values from ≤0.001 to 0.039), 3 channels for patients with MDD (*p*-values from 0.016 to 0.043) and 7 channels for patients with BPD (*p*-values from 0.044 to 0.007). Channels that did not show statistically significant differences in deoxy-haemoglobin between the pre-task baseline and task periods are in white.

**Supplementary table 2.** Associations between mean oxy-haemoglobin measured at channel 36 and clinical variables.

|  | HC | | MDD | | BPD | |
| --- | --- | --- | --- | --- | --- | --- |
|  | Test statistic | *p*-value | Test statistic | *p*-value | Test statistic | *p*-value |
| Number of words ^a^ | -0.362 | 0.059 | -0.049 | 0.795 | 0.206 | 0.274 |
| Family psychiatric history ^b^ | 1.35 | 0.192 | -0.756 | 0.456 | 1.2 | 0.243 |
| GAF score ^a^ | 0.155 | 0.431 | 0.101 | 0.587 | 0.054 | 0.778 |
| HAM-D score ^a^ | -0.231 | 0.237 | -0.124 | 0.507 | 0.135 | 0.478 |
| BPQ score ^a^ | - | - | -0.066 | 0.724 | -0.12 | 0.527 |
| Age at illness onset (years) ^a^ | - | - | -0.215 | 0.246 | -0.337 | 0.074 |
| Duration of illness (years) ^a^ | - | - | -0.212 | 0.252 | -0.267 | 0.162 |
| Past admission to psychiatric ward ^b^ | - | - | -1.14 | 0.263 | -0.404 | 0.689 |
| Pharmacotherapy ^b^ | - | - | 0.423 | 0.675 | -1.269 | 0.29 |
| Fluoxetine eq. dose (mg/day) ^a^ | - | - | 0.164 | 0.477 | -0.09 | 0.683 |
| Diazepam eq. (mg/day) ^a^ | - | - | -0.564 | 0.322 | -0.797 | 0.203 |
| Chlorpromazine eq. dose (mg/day) ^a, c^ | - | - | - | - | -0.539 | 0.168 |
| Mood stabiliser ^b, c^ | - | - | - | - | -1.439 | 0.167 |

^a^ Pearson’s correlation test.

^b^ Student’s t-test.

^c^ 2 patients with major depressive disorder were on antipsychotics, and 1 patient with major depressive disorder was on a mood stabiliser. Correlation analysis between antipsychotic equivalent dose and mean oxy-haemoglobin, as well as a comparison of mean oxy-haemoglobin of patients receiving mood stabilisers and those who were not could not be done for this diagnostic group.
